# City lizards are more social

**DOI:** 10.1098/rsbl.2025.0326

**Published:** 2025-09-24

**Authors:** Avery L. Maune, Tobias Wittenbreder, Duje Lisičić, Barbara A. Caspers, Ettore Camerlenghi, Isabel Damas-Moreira

**Affiliations:** ^1^ Department of Behavioural Ecology, Bielefeld University, Bielefeld, North Rhine-Westphalia, Germany; ^2^ Department of Biology, University of Zagreb, Zagreb, Croatia; ^3^ Joint Institute for Individualisation in changing Environments (JICE), University of Münster and Bielefeld University, Bielefeld, Germany; ^4^ Collegium Helveticum, Zürich, Switzerland

**Keywords:** urban ecology, *Podarcis muralis*, social networks, reptile, sociality, rural

## Abstract

Cities are expanding globally, exposing animals to novel ecological conditions that can alter the frequency and nature of their social interactions. Urban habitat features, such as built infrastructure and patchy resource distributions, can constrain movement and promote aggregation, potentially increasing encounters among conspecifics and introducing unique social challenges. Understanding how urbanization affects social behaviour is therefore crucial. However, these effects remain poorly understood, and studies on solitary or non-gregarious species are particularly scarce. Here, we investigate how urbanization influences social behaviour in the common wall lizard (*Podarcis muralis*), a territorial species and successful urban dweller. We constructed social networks using proximity-based association data from urban and non-urban lizard populations. Urban lizards had more social connections, stronger associations and were observed in more associations overall. These differences were not explained by variation in population density. We propose that spatial constraints and resource heterogeneity in urban habitats may enhance social tolerance. Our results reveal that urbanization can reshape social behaviour even in less gregarious species and suggest that shifts in social strategies may facilitate persistence in urban landscapes.

## 1. Introduction

Cities are major drivers of environmental change worldwide. Over half of the global human population currently lives in urban areas, a figure projected to increase to 68% by 2050, when the total population is also expected to reach 9.7 billion people [1,2]. As urban areas expand alongside the growing human population, transforming natural habitats, it is increasingly urgent to understand how urbanization affects wildlife. Urban environments introduce novel ecological conditions, including habitat fragmentation, altered resource distributions, sensory pollutants, novel predators and increased human activity [3]. As a result, animals in urban areas often adjust their behaviour to cope with these highly modified environments [4,5], including changes at the social level.

Urbanization can reshape the ecological context of interactions, exposing animals in cities to distinct selection pressures on social behaviour [6,7]. Urban habitat features can alter the frequency, intensity and outcomes of social encounters [8–10]. For example, buildings, roads and other artificial structures in cities can fragment the habitat and alter movement patterns, potentially increasing conspecific density within habitat patches and consequently the likelihood of social encounters [11,12]. Spatially clumped anthropogenic resources can promote gregariousness in otherwise solitary species [13,14] or, conversely, intensify competition [15]. Differences in predator abundance or human presence may also influence social tendencies, as animals often adjust grouping behaviour in response to perceived threats [10,16]. Thus, environmental conditions in urban areas can reshape the costs and benefits of associating with conspecifics, potentially affecting the social structure of a population [17]. However, while the extent and direction of social responses to environmental change can vary depending on species’ life-history traits, social system and movement ecology [7,18,19], most research on the social consequences of urbanization has focused on highly social species and specific taxa, such as birds and mammals. How urbanization shapes social behaviour in species with more solitary tendencies, which may benefit from avoiding conspecifics, and likely face different social stressors in cities, is comparatively less well understood. In these species, spatial and movement constraints may limit the ability to avoid conspecifics, potentially forcing individuals into larger aggregations or more stable social groupings.

Reptiles can be severely impacted by anthropogenic habitat modification [20,21]. The effects of urbanization on their social behaviour, however, are relatively unexplored [22,23], despite some species being common urban dwellers. Reptiles differ from many other vertebrates in their energetic requirements and movement ecology and often reduce their home range in response to urbanization [24], which can in turn shape their social environment [19]. Here, we examine how urbanization affects social structure in the common wall lizard (*Podarcis muralis*), a territorial species that can successfully inhabit highly modified urban environments. In this species, social interactions primarily occur in the context of competition for access to resources and mates [25]. Given that urban areas can be severely fragmented, likely reducing suitable habitat space and restricting dispersal for lizards, we hypothesize that urban habitats will promote more frequent social encounters. We predict that urban populations will have higher population density and that urban lizards will be more socially tolerant, reflected by greater social connectivity and stronger social associations. To test this, we used proximity data to construct social networks for common wall lizard populations in urban and non-urban habitats.

## 2. Methods

### (a) Data collection

We studied urban and non-urban populations of the common wall lizard in Croatia. Within each habitat, we sampled three lizard populations along distinct transects, each 25 m long and spaced at least 350 m apart (electronic supplementary material, figure S1). Urban transects (P1–P3) were located within the city of Rovinj, a popular tourist destination characterized by high levels of human activity and a high proportion of built-up area. Non-urban transects (P4–P6) were located in Park Zlatni Rt, a coastal area comprised of forest and natural rocky outcrops, with more limited human disturbance and minimal infrastructure. Based on visual classification of satellite imagery, urban transects were primarily composed of built-up area (64–75%), including roads, pavement, buildings and walls, while non-urban transects were primarily composed of natural substrate (88–100%), including rocks and vegetation (electronic supplementary material, figure S2; details in the electronic supplementary material). The urban and non-urban areas are approximately 3 km apart, allowing us to control for natural variation in climatic conditions between locations. *Podarcis muralis* home range size is estimated to vary between 5 and 25 m^2^ [26], and we did not observe any individuals moving between transects. We estimated the population density at each transect by conducting line transect surveys and counting the number of individuals within 10 m of the observer [23]. We conducted 10 surveys for each transect on separate days, when the weather was optimal for lizard activity (i.e. sunny with a temperature above 23°C), with five surveys conducted in the morning and five in the afternoon.

For behavioural observations, lizards were first captured by noosing and held individually in breathable bags while being transported to a shaded area. We then recorded lizards’ snout–vent length, weight and individual photographs. Afterwards, animals were marked with two pieces of uniquely numbered cloth tape (TESA^®^, Hamburg, Germany) placed on the lizard’s back, which naturally came off when shedding [27]. All lizards were then released at their exact collection point, within 10 h of their capture.

Behavioural observations were conducted in June and July 2024 across a total of 21 observation days. We observed each transect twice daily in parallel between the urban and non-urban habitats. Observations lasted 30 min per transect and were conducted in the morning (08.00–11.30 h) and afternoon (15.00–17.30 h), corresponding with the lizard’s peak activity periods. We used binoculars to identify marked individuals and score social associations. In total, 94 lizards were marked and observed at least once across the observation days (table 1). We used spatial proximity as a proxy for social associations [28], a commonly used approach that can be applied to species that display cryptic social structures or engage in infrequent direct interactions [29]. We defined associations as individuals that were observed within 2 m of each other [30–32], a distance that is easily traversable and well within the visual range of common wall lizards for detecting conspecifics [33]. Distance was measured as the actual path a lizard would need to traverse, accounting for both horizontal and vertical separation. Associations were recorded regardless of vertical separation (e.g. one lizard on the ground and one lizard on a wall), provided that no physical barriers blocked direct access between the individuals (e.g. wall or dense vegetation). Only instances where both individuals were displaying tolerant behaviour (e.g. basking, foraging) were recorded as associations. Aggressive interactions (e.g. fighting, chasing, avoidance) were rare in both urban (*n* = 6; 1.6% of observations) and non-urban (*n* = 5; 2.9%) populations and were excluded from our dataset. Copulation events were also rare in urban (*n* = 4; 1.8%) and non-urban (*n* = 1; 0.6%) populations and were likewise excluded.

**Table 1.** Summary of (a) the number of lizards marked in each population and (b) the subset of lizards observed at least three times and included in the social network analysis. F, female; M, male; sub, sub-adult.

		(a) marked lizards (*n* = 94)				(b) social networks (*n* = 69**)**			
	population	*N*	M	F	sub	*N*	M	F	sub
urban	P1	11	6	4	1	9	4	4	1
	P2	36	16	15	4	27	13	10	4
	P3	16	9	5	2	11	6	5	2
non-urban	P4	12	8	3	0	9	6	3	0
	P5	10	7	3	0	5	5	0	0
	P6	10	7	3	0	8	6	2	0

### (b) Social network construction and statistical analysis

We constructed undirected, weighted social networks for each of the six populations. We excluded individuals that were observed less than three times and included a total of 69 lizards in our social networks (table 1). We quantified individual sociability using two network metrics: binary degree and weighted degree, using the *sna* package (v. 2.8) [34]. Binary degree refers to the number of unique connections a lizard has in the network, while weighted degree represents the sum of edge weights of a lizard and reflects the total social bond strength an individual has with all of its connections (i.e. the frequency of associations with each connection).

Before proceeding with the data analysis, we confirmed that the observation rate did not differ between habitats (electronic supplementary material, table S1), indicating that observation effort was consistent between urban and non-urban transects. To test whether urbanization predicts social behaviour, we compared binary degree (i.e. number of social connections) and weighted degree (i.e. strength of social associations) across habitats using two GLMMs. We modelled (a) binary degree with a Poisson distribution and (b) weighted degree with a Tweedie distribution, which can accommodate right-skewed, zero-inflated data. For both models, we included habitat (urban, non-urban) and sex (female, male, sub-adult) as fixed effects. Transect (P1–P6) was included as a random effect to account for variation among populations. Data stream permutations that permute network edges are commonly used to control for non-independence in social network data [35]; however, our non-urban networks contained few edges and lacked sufficient structure to effectively run a data stream permutation. Consequently, to provide further support that observed differences in network structure across habitats were not due to chance, we ran another GLMM that compared (c) the total number of associations observed per marked lizard (*n* = 94), between habitats. As in the models above, we included habitat and sex as fixed effects and transect as a random effect. This response variable (c) included all observed associations for each marked lizard, with both marked and unmarked individuals, without a minimum observation threshold. This approach allowed us to account for potential differences in the proportion of marked individuals between habitats, as not all lizards in the study sites could be captured and marked. Consequently, a marked lizard that is frequently associated with an unmarked individual could appear to have no social connections in the network (a and b), but these interactions would be captured in the association count (c). By comparing association counts across habitats, we were able to assess whether our network-based results reflected true differences in social behaviour, rather than artefacts of variation in marking success. We originally modelled this third GLMM (c) with a Poisson distribution, but diagnostic tests indicated significant overdispersion, so we refitted the model with a negative binomial distribution. Finally, to understand whether potential differences in sociality across habitats could be confounded by variation in lizard population densities, we modelled the number of lizards observed per survey using a GLMM with a Poisson distribution (d), including habitat (urban, non-urban) as a fixed effect and transect (P1–P6) as a random effect. GLMMs were fitted using the packages *lme4* (v. 1.1.37) [36] and *glmmTMB* (v. 1.1.11) [37]. Model assumptions were checked using the performance package (v. 0.15.0) [38]. All analyses were conducted in R (v. 4.4.2) [39].

## 3. Results

**Figure 1.**
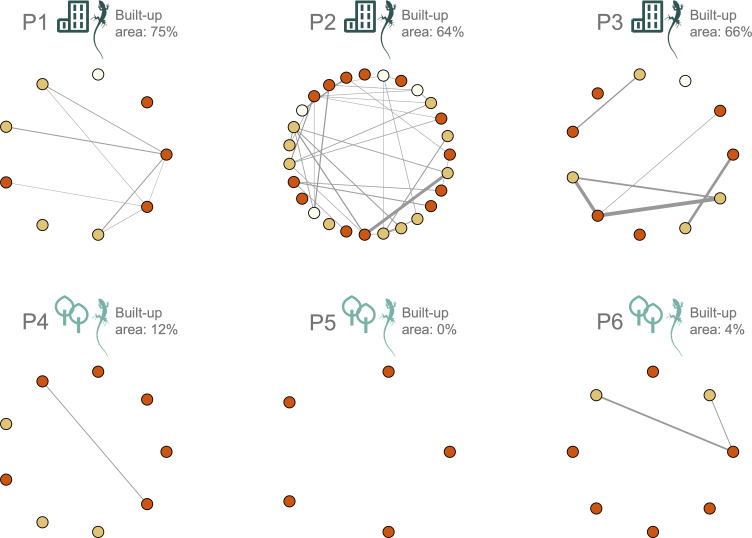
Social networks for urban (P1–P3) and non-urban (P4–P6) lizard populations. Each node represents an individual lizard and edge thickness indicates the frequency of observed associations between pairs. Node colour represents sex: dark orange = males, pale yellow = females and white = sub-adults. Networks were visualized using the *igraph* package (v. 2.1.4) [40] in R.

**Figure 2.**
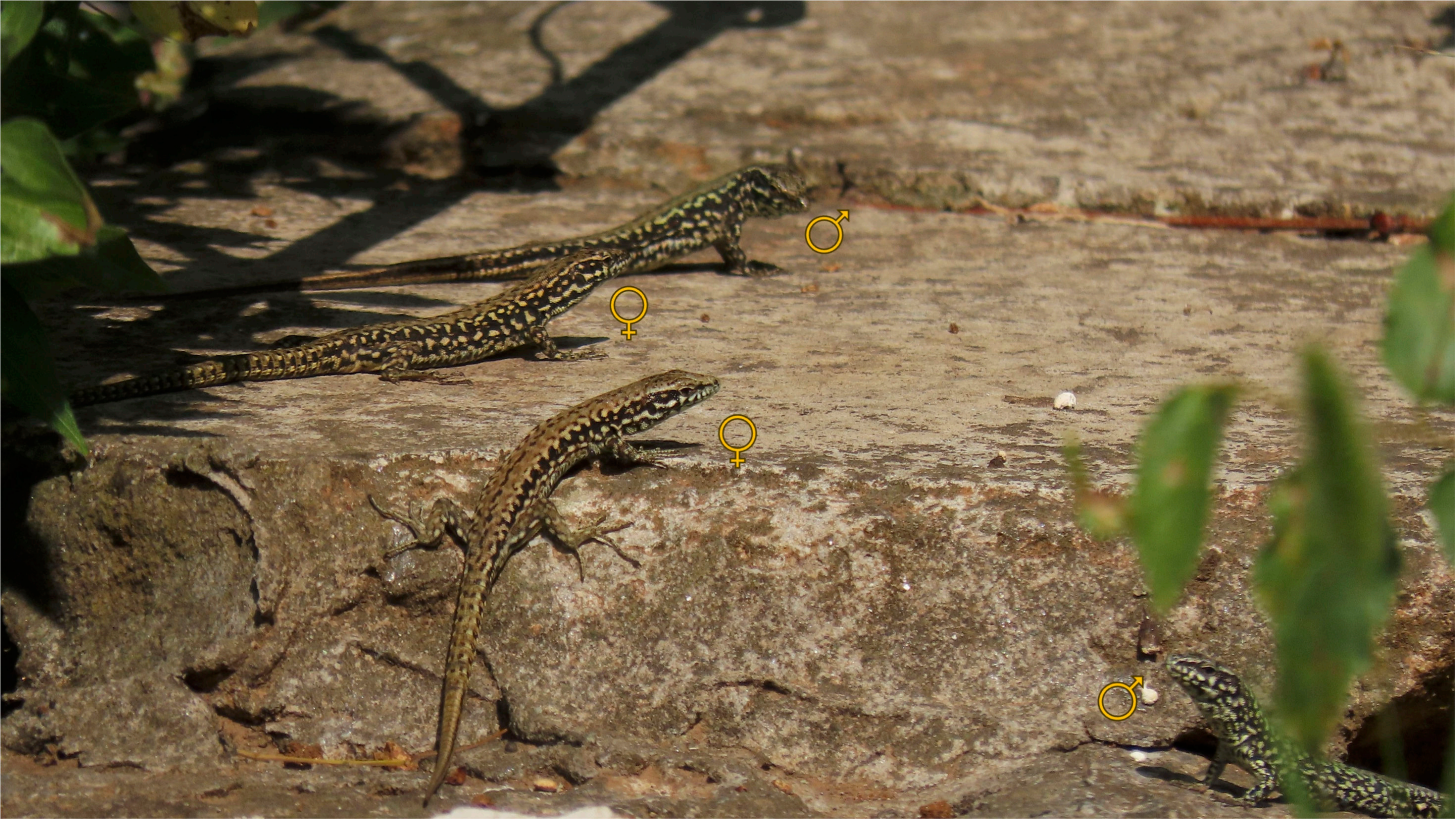
Common wall lizards (*Podarcis muralis*) in an urban habitat. This photo shows two unmarked males and two unmarked females basking on a stone wall, illustrating the close proximity typical of associations observed in urban areas. These individuals were photographed outside the study period and were not marked or included in the dataset.

**Figure 3.**
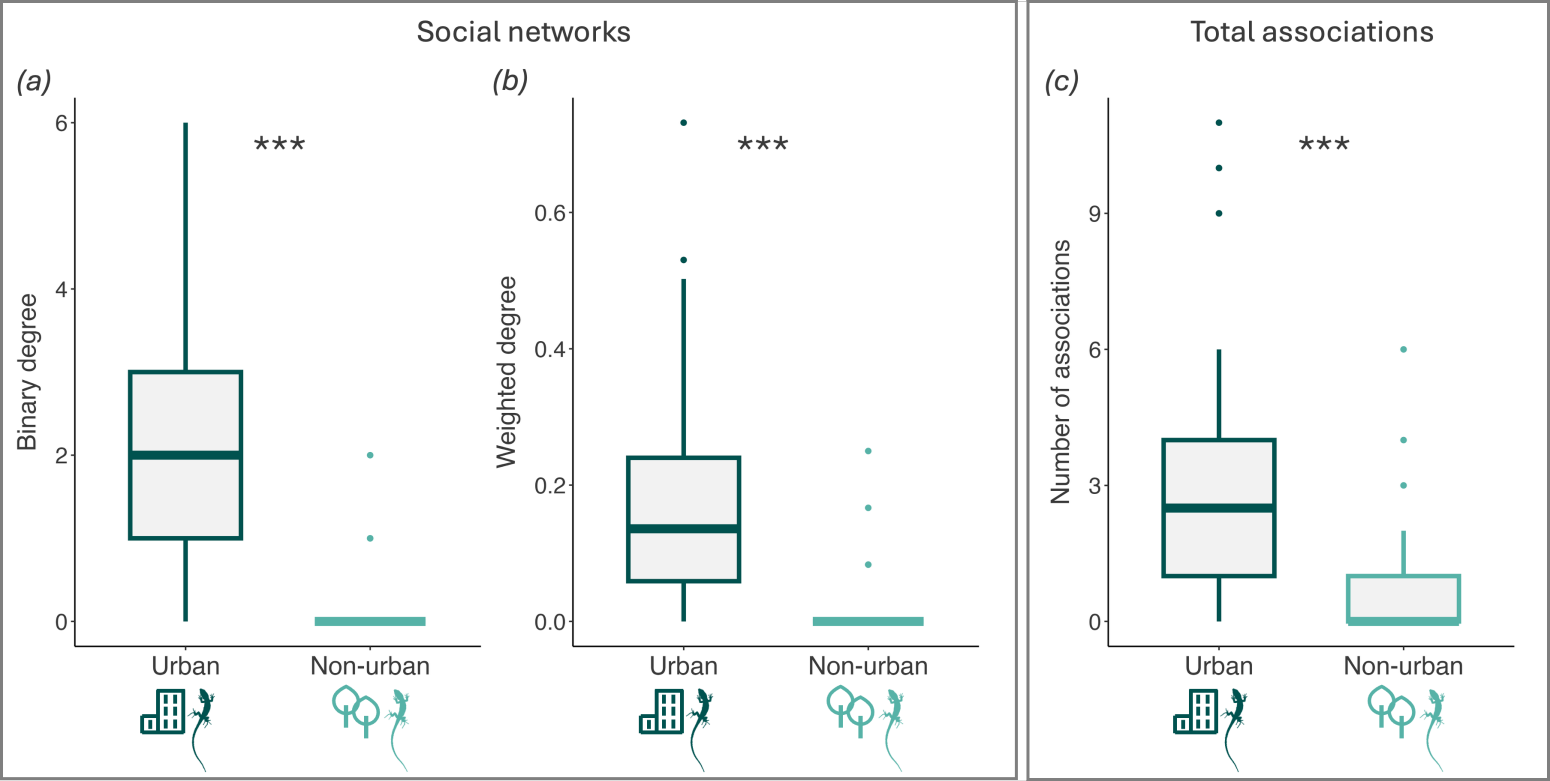
Boxplots comparing (*a*) binary degree, (*b*) weighted degree and (*c*) the number of total associations between urban and non-urban common wall lizards. Social network metrics (*a,b*) were calculated only for individuals observed at least three times, with associations recorded exclusively between marked individuals (*n* = 69). The total association count (*c*) included all marked individuals (*n* = 94) and included associations with both marked and unmarked lizards. Boxes represent the interquartile range, bold lines indicate medians, and whiskers extend to the minimum and maximum values. Asterisks denote significance (***p < 0.001).

From our social network analysis (figure 1), 81% (38/47) of urban individuals were connected to the network, while only 23% (5/22) of non-urban individuals were connected to the network (i.e. they were observed in association with another marked lizard during our study; see figure 2 for an example of an association). Urban lizards had a significantly higher binary degree (a) (table 2; figure 3*a*) and weighted degree (b) than non-urban lizards (table 2; figure 3*b*). On average, urban lizards had 1.9 (±0.2) social connections, while non-urban lizards only had 0.3 (±0.1). When including associations between marked and unmarked individuals (c), marked urban lizards were also observed in more associations (table 2; figure 3*c*). Among marked individuals, urban lizards were observed on average in 3.3 (±0.4) associations, while non-urban lizards were observed in only 0.8 (±0.2) associations. On average, urban lizards were in association during 53% of observations, while non-urban lizards were in association during 15% of observations. While we found a non-significant trend for higher population density (d) in the urban habitat (GLMM, β = −0.572 ± 0.317, *z* = −1.802, *p* = 0.07; electronic supplementary material, table S2), this was primarily driven by one more dense urban population (P2; electronic supplementary material, figure S3). Even when excluding this population from our analysis, we still found consistent significant differences in (a) binary degree, (b) weighted degree and (c) the total number of associations, suggesting that population density is not solely driving observed differences (electronic supplementary material, table S3).

**Table 2.** Outcomes of GLMMs for (a) binary degree, (b) weighted degree and (c) total associations. All significant results are highlighted in bold.

	estimate	s.e.	z-value	*p*‐value
**(a) binary degree** ***N*** **= 69**				
intercept (urban: female)	0.615	0.234	2.630	**0.009**
habitat (non-urban)	−1.848	0.479	−3.857	**<0.001**
sex (male)	−0.140	0.219	−0.640	0.522
sex (sub-adult)	−0.167	0.342	−0.490	0.624
**(b) weighted degree** ***N*** **= 69**				
intercept (urban: female)	−1.514	0.184	−8.234	**<0.001**
habitat (non-urban)	−1.724	0.425	−4.058	**<0.001**
sex (male)	−0.355	0.258	−1.379	0.168
sex (sub-adult)	−0.616	0.462	−1.334	0.182
**(c) total associations** ***N*** **= 94**				
intercept (urban: female)	1.009	0.226	4.462	**<0.001**
habitat (non-urban)	−1.314	0.301	−4.360	**<0.001**
sex (male)	0.174	0.233	0.750	0.453
sex (sub-adult)	0.235	0.396	0.592	0.554

## 4. Discussion

We found significant differences in social behaviour between urban and non-urban common wall lizards (*P. muralis*). Urban lizards had more social connections and stronger associations, suggesting a shift towards increased social tolerance in urban environments. Notably, these differences could not be explained by variation in population density, indicating that other urban habitat features are likely shaping patterns of social behaviour in this species.

In our study, most urban lizards were socially connected, while the majority of non-urban lizards had no detectable social connections, suggesting a fundamental difference in social structure between habitats. Differences in the spatial and temporal distribution of habitat components intrinsic to urban environments likely influence movement patterns and opportunities for social interactions [18,19]. This is because in cities, animals are often confined to smaller, isolated habitat patches that are surrounded by built infrastructure, which can restrict dispersal and limit opportunities to avoid conspecifics. By increasing the frequency of repeated encounters, these spatial constraints may select for reduced aggression and greater tolerance among familiar neighbours [41]. In addition, urban environments can have more fine-scale habitat heterogeneity, with key resources such as refuges, food and basking sites distributed in a clumped and patchy manner across space and time [42,43]. For example, anthropogenic structures in cities, such as stone walls, can provide abundant crevices and microhabitats for small lizards [44], increasing opportunities for spatial overlap and aggregation [45]. Clumped or patchy resource distributions can reduce the benefits of excluding conspecifics [46]. Instead, social tolerance may be favoured as it allows individuals to access resources while avoiding costly conflicts [47].

Adjusting behaviour in response to changing social conditions is likely an important factor in allowing some species to cope with urban life. For territorial species, space is a limiting resource [48], and the built-up environment can reduce the feasibility of defending exclusive territories. Becoming more tolerant to the presence of conspecifics may represent a behavioural shift that allows animals to adjust to the ecological conditions of urban habitats. Increased social tolerance could mitigate the energetic and physiological costs associated with direct interactions [9,22] and facilitate access to social information, which could be further advantageous in navigating complex or unpredictable urban landscapes [3]. This shift may ultimately lead to the emergence of alternative social strategies, such as shifts from territorial behaviour to dominance hierarchies [26,49].

Species with limited dispersal abilities can offer valuable insights into how environmental variation shapes social structure. In urban environments, restricted dispersal may lead to increased kin structuring within habitat patches, potentially reinforcing tolerance through higher relatedness [50]. Stable social associations, based on familiarity or relatedness, can reduce the costs of territoriality and enhance fitness [51]. However, restricted dispersal can increase the risk of inbreeding, and shifts towards social tolerance may also carry important fitness consequences, especially in species like the common wall lizard, where territoriality plays a central role in accessing resources and attracting mates [25]. Individual variation in social connectedness could potentially increase reproductive variance, affecting sexual selection regimes. In addition, higher contact rates in more connected urban networks may facilitate pathogen and parasite transmission [52], potentially increasing immune costs, which can negatively impact health and reproduction.

Our results demonstrate the substantial impact that urbanization can have on social behaviour, even in species that are on the lower end of the sociality spectrum. Understanding how social systems respond to anthropogenic environments is crucial, as social interactions play a key role in resource access, the spread of information and disease, and ultimately fitness.

## Data Availability

All data, code, and metadata are available from the Dryad Digital Repository [53]. Supplementary material is available online [54].
